# Dressed-photon–phonon (DPP)-assisted visible- and infrared-light water splitting

**DOI:** 10.1038/srep04561

**Published:** 2014-04-02

**Authors:** Takashi Yatsui, Tsubasa Imoto, Takahiro Mochizuki, Kokoro Kitamura, Tadashi Kawazoe

**Affiliations:** 1 Department of Electrical Engineering and Information Systems, School of Engineering, The University of Tokyo, Tokyo 113-8656, Japan; 2 Department of Mechanical, Electrical and Electronic Engineering, Shimane University, Shimane 690-8504, Japan

## Abstract

A dressed-phonon–phonon (DPP) assisted photocatalyst reaction was carried out to increase the visible light responsibility, where the photon energy of the radiation, which ranged from visible to infrared light is less than band gap energy of the photocatalyst (ZnO, 3.3 eV). The dependence of the photocurrent on excitation power indicated that two-step excitation occurred in DPP-assisted process. A cathodoluminescence measurement also supported the conclusion that the visible- and infrared-light excitation originated from DPP excitation, not from defect states in the ZnO nanorod photocatalyst.

Hydrogen has been attracting much interest as a clean energy source. For example, photo-electrolysis of water using photocatalysts such as TiO_2_ as the photoelectrode under solar light irradiation is a promising means of generating hydrogen without generating CO_x_^1^. Although TiO_2_ has great potential as photocatalytic material, only the ultraviolet light in the solar spectrum can be utilized by TiO_2_ because of its wide band gap (3.3 eV). To increase the efficiency of solar light utilization, photocatalytic materials that are activated with light in the visible region have been studied. Conventional approaches to the realization of a visible light photocatalyst include exploring new materials with smaller band gaps or creating new states within the band gap by introducing dopants or defects^2^,^3^,^4^,^5^. In this paper, we introduce a different approach to the visible light photocatalysis; the dressed-photon–phonon (DPP) assisted process^6^. Semiconductor materials have phonon excited sates within the band gap. However, propagating far-field light cannot excite electrons from the valence band to these phonon excited states, because these transitions are electric-dipole-forbidden. However, a DPP can be generated in nanometre-sized semiconductor materials, and this DPP can excite multiple modes of coherent phonons around nanometer-sized structures. Thus, the DPP can activate the dipole-forbidden phonon transition^7^,^8^. In the DPP-assisted process, two-step excitation from the valence band to the conduction band is realized via an intermediate phonon state, and the energy required to create electron-hole pairs is therefore smaller than the band gap energy.

The phonon-assisted two-step excitation can be explained as follows (see Fig. 1). In the first step (I), the transition from the ground state 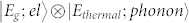, which is the initial state, to an intermediate state 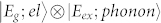 occurs, Here, the ket vector 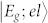 represents the electron ground state, and 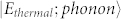 and 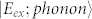 respectively represent the phonon excitation state that is determined by the crystal lattice temperature and the photon excitation state that is determined by the photon energy. The symbol 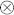 represents the direct product of the ket vectors representing the two state. Because it is an electric-dipole-forbidden transition, an optical near field is essential for this excitation.

Second, excitation proceeds from the intermediate state 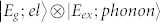 to the final state 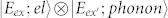 (step (II)). Here 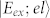 represents the electron excitation state, and 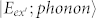 represents the phonon excitation state. Since this is an electric-dipole-allowed transition, the excitation occurs not only via the DPP but also via propagating light. After this excitation, the phonon excitation state relaxes to a thermal equilibrium state with an occupation probability determined by the lattice temperature, and the process ends with excitation to the electron excitation state 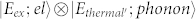.

Based on the DPP-assisted process, we developed applications including photochemical vapour deposition^7^, photolithography^9^, excitation of dye^10^, and photovoltaic devices^11^. Note that the DPP-assisted two-step excitation is different from conventional two-photon excitation, because the intermediate excite states can be real states in the DPP-assisted process, while the intermediate state is virtual in conventional two-photon excitation. Here, we applied the DPP-assisted process to the visible- and infrared-light photocatalytic reaction using a nano-structured photocatalytic electrode.

## Results

### Fabrication of a nanostructured electrode and evaluation of its optical properties

We employed ZnO nanorods with nanometer-scale tip diameters as the nano-structured photocatalytic electrode. Electrolysis of water was performed using these ZnO nanorods as a working photoelectrode to evaluate the DPP-assisted photocatalytic process. Using catalyst-free metal-organic vapour phase epitaxy (MOVPE), the ZnO nanorods were grown on a sapphire (0001) substrate^12^. Prior to the ZnO nanorod deposition, the sapphire substrate was coated with an indium tin oxide (ITO) thin film to increase in conductivity. The thickness of the ITO film was approximately 200 nm. Diethylzinc (DEZn) and oxygen (O_2_) were used as the reactant gases, and Ar was used as the carrier gas in the MOVPE process. The pressure inside the reactant chamber was maintained at 5 Torr. The substrate temperature was controlled using a thermocouple and a radio-frequency-heated carbon susceptor. In the MOVPE process, the diameter of the ZnO nanorods was determined by the growth temperature^13^. Two electrodes were prepared for measurement: one electrode with thick ZnO nanorods (100 nm nanorods) with diameters of 100 nm and another electrode with fine ZnO nanorods (20 nm nanorods) with an average diameters of 20 nm. Scanning electron microscopic (SEM) images of the 100 nm and 20 nm nanorods are shown in Figs. 2(a) and 2(b), respectively.

Since it has been reported that crystal defects such as impurities or oxygen deficiencies enhance the visible photocatalytic activity^4^,^5^, we excluded this possibility by studying the optical properties of ZnO nanorods electrodes and the ZnO bulk substrate to examine the crystal defects. Figure 2 (c) shows a high-resolution transmission electron microscopy (TEM) image of the area show in Fig. 2(b). Figures 2(d) and 2(e) show the cross sectional profiles along the d–d′ and e–e′ lines of the area shown in Fig. 2(c), revealing that the ZnO nanorods consisted of single crystals with lattice spacings of 0.498 and 0.283 nm. These values correspond to crystalline wurtzite ZnO with lattice constants of *c* = 0.521 nm and *a* = 0.325 nm^14^. The TEM image also revealed that nanorods grew along the *c*–axis. As a reference ZnO material, a commercially available ZnO bulk substrate grown by hydrothermal synthesis and a sputtered ZnO film were employed. The surface roughness, *R_a_*, values of the ZnO bulk substrate and the sputtered ZnO film were 0.21 nm and 6.63 nm, respectively.

The chathodoluminescence (CL) spectra of the ZnO electrodes are shown in Fig. 3(c). The CL spectral shapes were similar. The peak around 3.3 eV was attributed to the emission from band-edge of ZnO (*I_BE_*), and peaks around 2.6 eV were attributed to the emission from the crystal defect levels due to oxygen vacancies, well known to appear as green luminescence^15^,^16^,^17^. The CL intensities from defect levels were higher for the ZnO bulk and the sputtered ZnO film than of the nanorods. The ratios of the CL intensity at visible light energies (*hν* ~ 2.6 eV) to that at the band-edge energy (*hν* = 3.3 eV) were 0.02 for bulk substrate, the sputtered film, and 100 nm nanorods, and less than 0.001 for the 20 nm nanorods. These results exclude the possibility of multi-step excitation via vacancies sites.

Prior to the DPP-assisted photocurrent generation, we measured dependences of the electrode potential for a photon energy of 4.66 eV, which is higher than the band gap energy of ZnO (Fig. 4(a)), and an input laser power of 300 μW. The results obtained confirmed that the flat band of all the ZnO electrodes occurred at approximately −0.7 V (vs. Ag/AgCl); this corresponds to the reported values of −0.7 to −0.9 V (vs. Ag/AgCl)^18^,^19^. Since a stable photocurrent was obtained at an electrode potential of 0.5 V (vs. Ag/AgCl) under low incident-light power, we set the electrode potential to 0.5 V (vs. Ag/AgCl) during the experiment to determine the dependence of the photocurrent on the incident-light power. Figure 4(b) shows the dependence of the photocurrent on the laser power during UV laser irradiation with a photon energy of 4.66 eV and an electrode potential of 0.5 V (vs. Ag/AgCl). From these results, we plotted the best-fit line for each samples, and all curves showed linear dependence of the photocurrent on the laser power. In addition, although the nanorod electrodes have a much larger surface area because of the small nanorod diameters, the value of the photocurrent was comparable to those of the bulk substrate with a flat surface, meaning that the changes in photocurrent generation due to the different surface areas should be negligible. Since almost all carriers were excited by photons with energies greater than the band gap energy, the DPP effect should be negligible for this photocurrent generation under UV light excitation.

### DPP-assisted photocurrent generation

**Figure 1:**
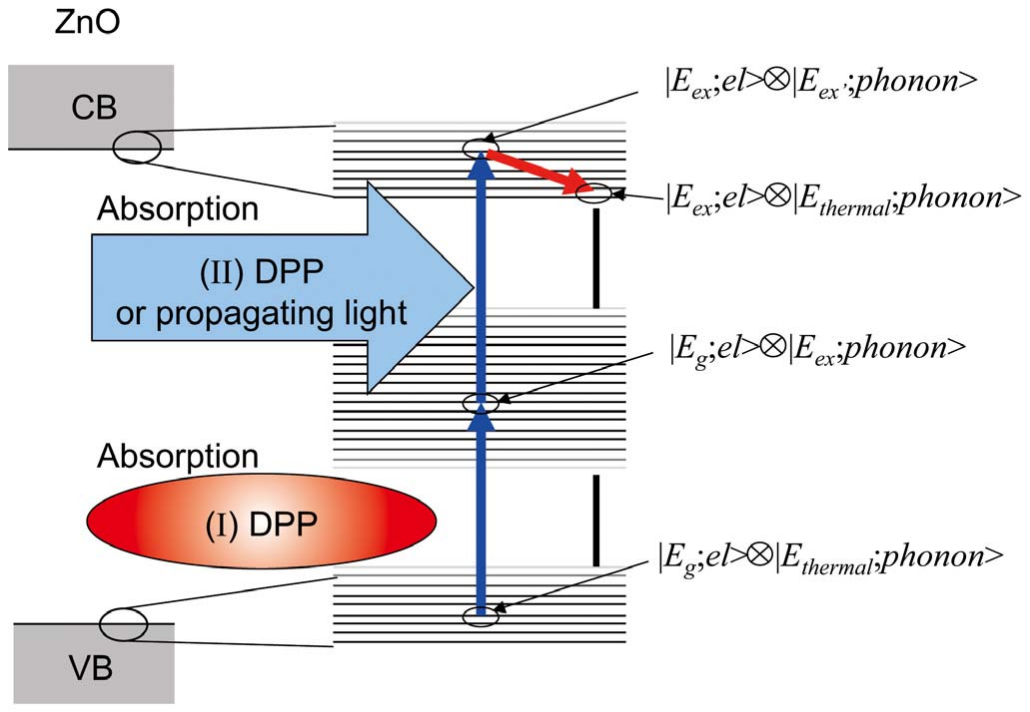
Concept of DPP-assisted carrier excitation. The direct product of the electronic state and the phonon state suggests that the eigenenergy of the electron-hole pair in the nanomaterials is modulated and has sidebands, which is the dual relation for the modulation of the DPP eigenenergies. These modulation sidebands correspond to an infinite number of phonon states. The many grouped horizontal lines represent these phonon states.

**Figure 2:**
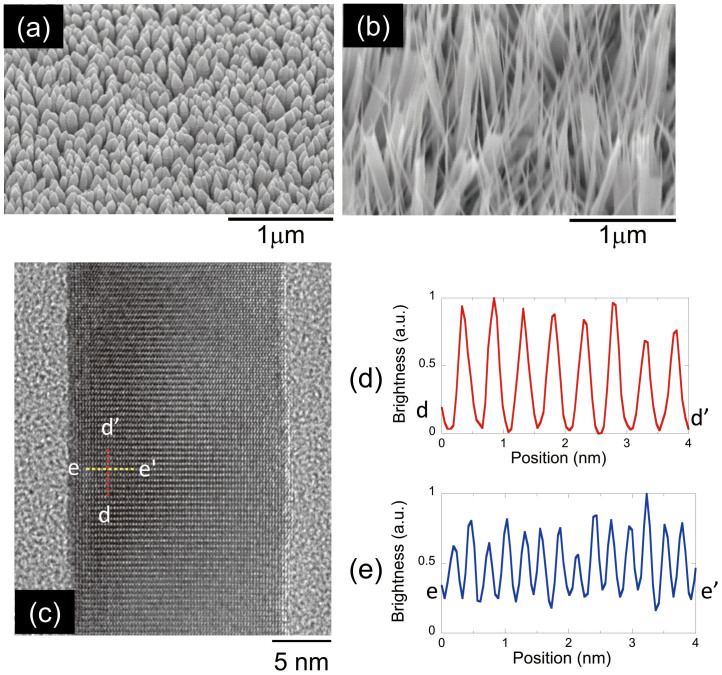
Nanorod properties. SEM images of (a) 100 nm nanorods and (b) 20 nm nanorods and (c) TEM image of the 20 nm nanorods. Figures 2(d) and (e) show ross-sectional profiles along the dashed lines d–d′ and e–e′, respectively, in the area shown in Fig. 2(c).

**Figure 3:**
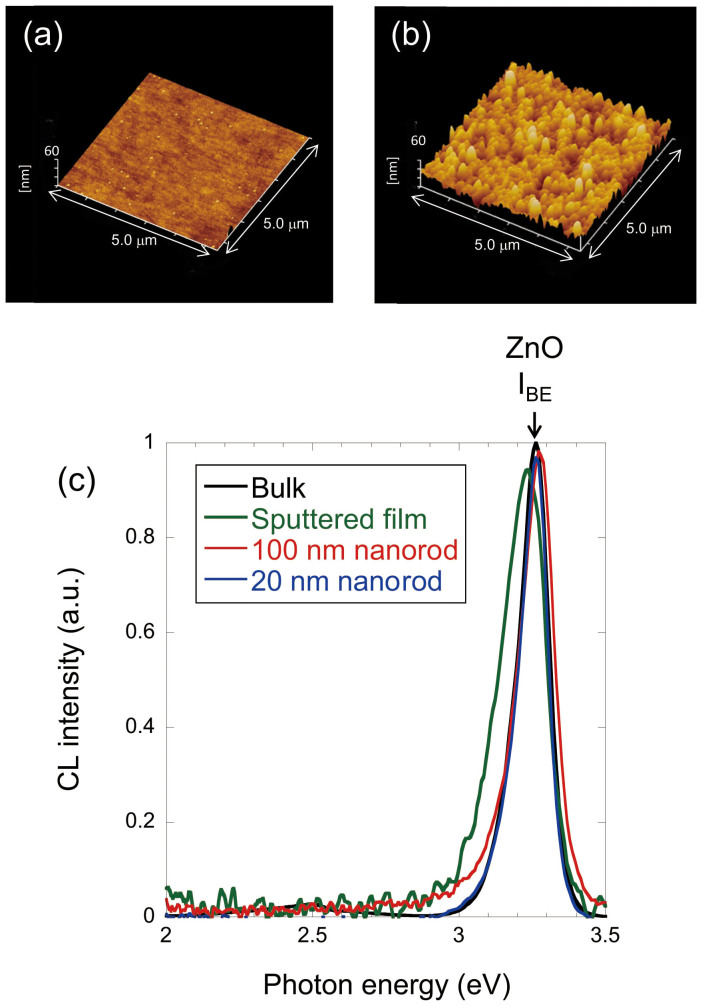
ZnO electrode properties. Atomic force micrography images of the (a) ZnO bulk substrate and (b) sputtered ZnO film. (c) The black solid curve is the CL spectrum of the bulk substrate, the red solid curve is the spectrum of the 100 nm nanorods, and the blue solid curve is the spectrum of the 20 nm nanorods.

**Figure 4:**
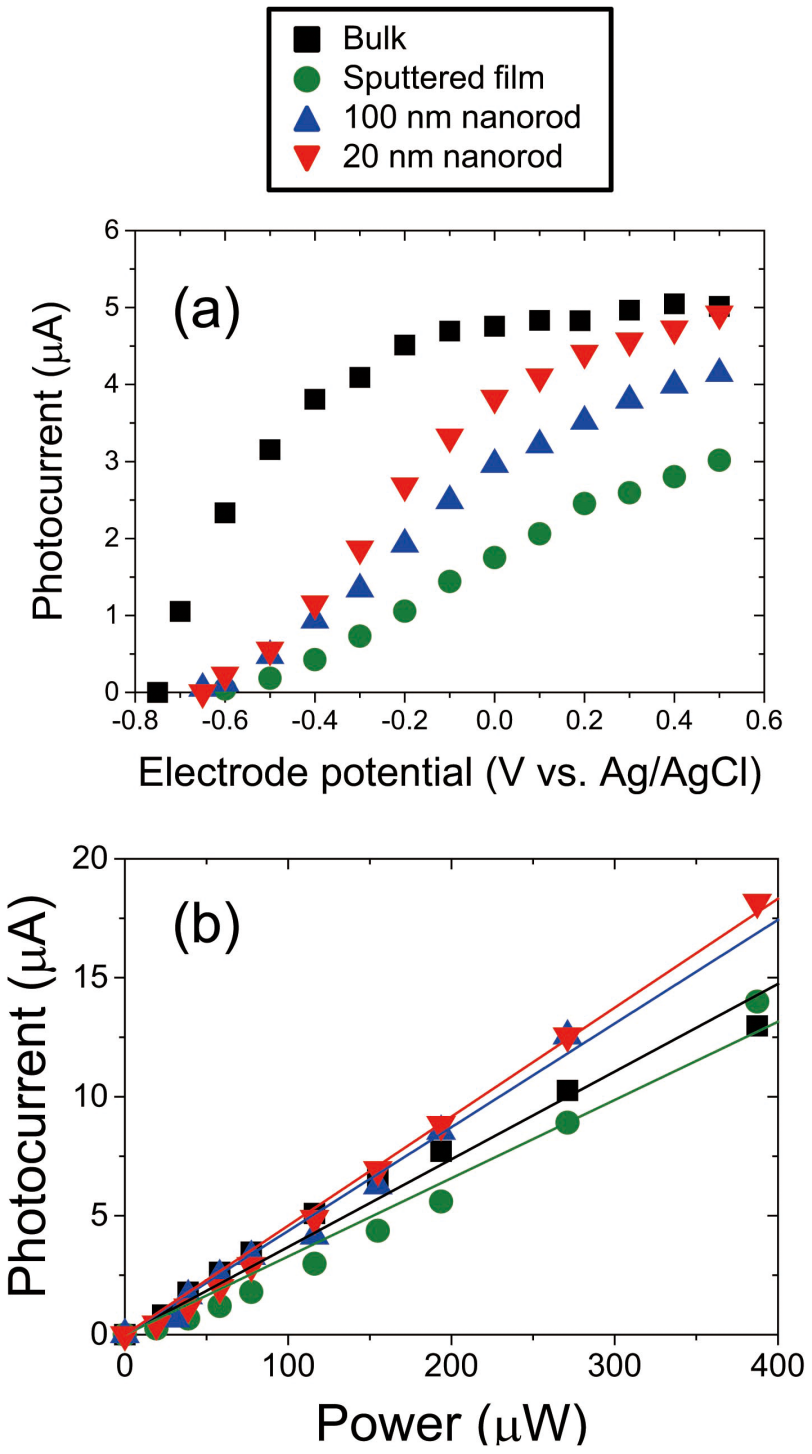
Dependence of photocurrent on (a) electrode potential and (b) UV laser (4.66 eV) power. In (b), electrode potential was set to 0.5 V (vs. Ag/AgCl). Solid lines in (b) were fitted assuming a linear dependence.

**Figure 5:**
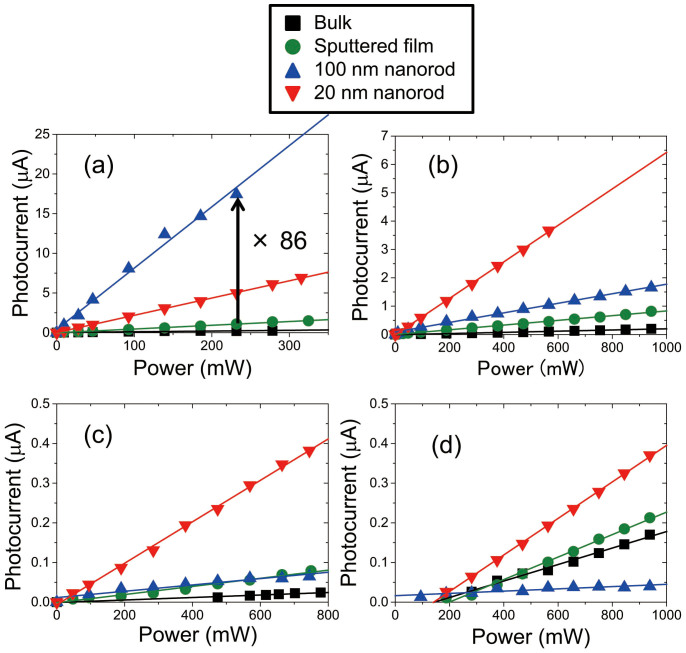
Dependence of current on laser power. Results for irradiation energies of (a) 2.62 eV (λ = 473 nm), (b) 2.33 eV (λ = 532 nm), (c) 1.85 eV (λ = 671 nm), and (d) 1.53 eV (λ = 808 nm). The electrode potential was set to 0.5 V (vs. Ag/AgCl). Solid lines were fitted assuming linear dependence.

We next measured the dependence of the photocurrent on the laser power under visible-light (*hν* = 2.62, 2.33, and 1.85 eV) (see Figs. 4(a), 4(b), and 4(c)) and infrared light (*hν* = 1.53 eV) laser irradiation (see Figs. 4(d)); in these cases, the photon energy was lower than the band gap energy. Although small photocurrent was detected for the bulk ZnO substrate and sputtered film, larger photocurrents were detected for both 100 nm and 20 nm nanorods electrodes. In the results for all laser excitation, a linear dependence of the photocurrent on laser power was observed. In the case of an excitation energy of 2.62 eV (Fig. 5(a)), the photocurrent generated in the 100 nm nanorods was 86 times higher than that in the bulk substrate. Such linear power dependences can be considered to arise as follows. In the multiple-photon excitation explained in Fig. 1 the excitation rate for the second-step excitation (step (II)) should be much larger than that for the first-step excitation (step (I)), because the second-step excitation is also induced by propagating far-field light since the transition from the intermediate state is optically allowed. Therefore, the second-step excitation process can occur once the first-step excitation process has completed, so the final photocurrent should have a linear power dependence. Note that a laser power density on the order of W cm^−2^ is 10^15^ times smaller than that used in multiple-photon processing with an ultra-short-pulse laser^20^. Therefore, the DPP-assisted excitation process did not originate from a conventional multiple-photon excitation process^21^.

**Figure 6:**
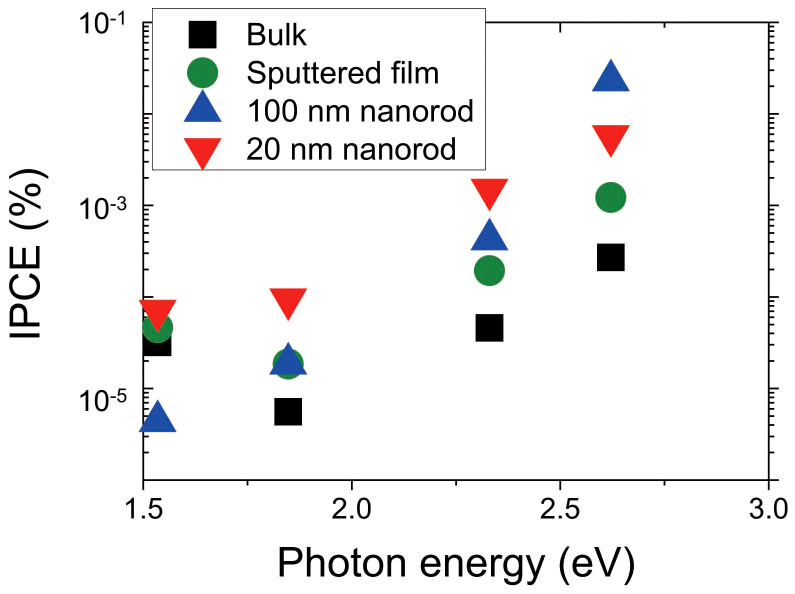
Dependence of incident-photon-to-current conversion efficiency (IPCE) on excitation photon energy.

To evaluate the dependence of the photocurrent on the incident photon energy, the incident-photon-to-current conversion efficiency (IPCE) were determined (see Fig. 6). That the value of IPCE for the bulk substrate at 1.53 eV (808 nm) was higher might be attributed to absorption of water in the near-infrared region^22^. While the IPCE value of the 100 nm nanorods was the largest at 2.62 eV, the IPCE value of the 20 nm nanorods was the largest at all other excitation levels. The dependence of the photocurrent on the photon energy can be considered to arise as follows. When the excitation photon energy decrease, the number of carriers excited by conventional absorption also decreases. Therefore, the ratio of the number of carriers excited by DPP to the number of carriers excited by conventional absorption increases.

In summary, a DPP-assisted excitation process was carried out using ZnO nanorods with small diameters. From visible light to infrared light, photocatalytic reaction with smaller photon energy than the band gap energy of the semiconductor electrode was realized. CL measurement revealed that this reaction did not originate from defect states in the ZnO nanorods. This phonon-assisted excitation process can be applied not only ZnO but also for other semiconductor materials, which can realize the higher efficiency of hydrogen generation using sun light.

Such a DPP-assisted process can also be enhanced when the structure is fabricated using a DPP-assisted fabrication process^23^. Therefore, further increases in photocurrent generation are expected if ZnO nanorods can be synthesized using a DPP-assisted process.

## Methods

### Photocurrent measurement

**Figure 7:**
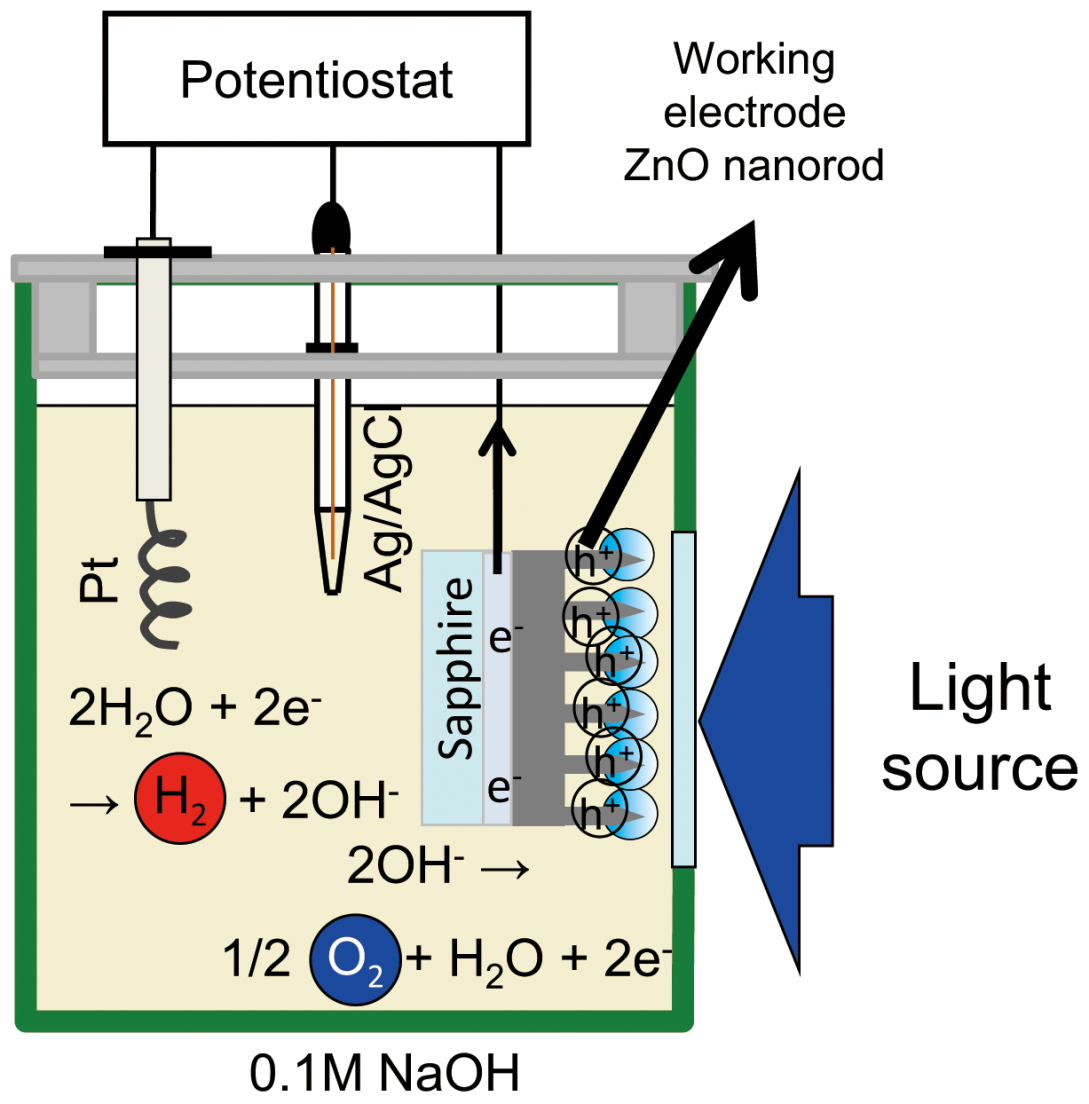
Schematic of photocurrent measurement. The DPP generated at the nanostructures can induce the DPP-assisted process. The photocatalytic water splitting performance is evaluated by performing a photoelectrochemical measurement with ZnO as the working electrode, Ag/AgCl as the reference electrode, and Pt as the counter electrode.

A schematic of the experimental setup is shown in Fig. 7. The counter and reference electrodes were a Pt wire and Ag/AgCl, respectively. To examine dependence of the photocurrent on the photon energy of the excitation light source relative to the band gap energy of ZnO (3.3 eV), a continuous wave (CW) He-Cd laser (photon energy *hν* = 3.8 eV) was used as an ultraviolet (UV) radiation source. In addition, as light sources, CW lasers (*hν* = 2.62, 2.33, 1.85, and 1.53 eV) were used. The diameter of the laser irradiation spot was approximately 1 mm. The electrolyte was 0.1 mol/L NaOH solution at room temperature. The lasers were irradiated for 20 s repeatedly at 20 s intervals. The value of the current was determined as the difference in the values before and after the laser was turned off. A potentiostat was used for the electrochemical measurements. The photoelectrochemical reaction rate was monitored as the current under laser irradiation. Hydrogen was generated at the Pt counter electrode, and oxygen was simultaneously generated by water oxidization and the decomposition of ZnO at the ZnO working electrode in basic solution^24^.

## Author Contributions

T.Y. planned the project. T.I., T.M. and K.K. performed experiments. T.K. was responsible for providing guidance for the experiments. All authors discussed the results. T.Y. wrote the manuscript. All authors reviewed the manuscript.
